# Cognitive and affective networks in patients referred for cognitive screening: latent profiles, bridge nodes, and exploratory directional structures

**DOI:** 10.3389/fpsyt.2026.1887588

**Published:** 2026-10-08

**Authors:** Yuting Wang, Yanqing Xie, Ling Yuan, Yanling Liu, Jinmei Zhang, Bixia Zhao, Fengying Zhang

**Affiliations:** 1 The Affiliated Nanhua Hospital, Sleep Medicine Center, Department of Neurology, Hengyang Medical School, University of South China, Hengyang, Hunan, China; 2 School of Nursing, The Affiliated Nanhua Hospital, Hengyang Medical School, University of South China, Hengyang, Hunan, China

**Keywords:** anxiety, bridge centrality, cognitive screening, depression, directed acyclic graph, latent profile analysis, Montreal Cognitive Assessment, network analysis

## Abstract

**Background:**

Lower cognitive performance and anxiety and depression frequently co-occur in patients referred for cognitive screening, but how the two domains are connected, and whether this differs across patient subgroups, is unclear. Prior network studies used abbreviated cognitive tools, self-report affective measures, and undirected models.

**Methods:**

In this cross-sectional study of 548 patients referred for cognitive screening at a Chinese general hospital, 16 network nodes were derived from the seven Montreal Cognitive Assessment domains, the two Hamilton Anxiety Rating Scale factors, and the seven factors of the 24-item Hamilton Depression Rating Scale. Gaussian graphical models with bridge expected influence were estimated for the overall sample and for each profile identified by latent profile analysis. Bayesian directed acyclic graphs with bootstrap resampling explored the direction of cross-domain associations in the overall sample and within each profile. The network comparison test compared profiles.

**Results:**

Cognitive and affective nodes formed two nearly completely separated communities (normalized mutual information = 1.000), connected mainly through language and anxiety/somatization, the two primary bridge nodes. Latent profile analysis identified three profiles: low cognitive performance (*n* = 114), high affective burden (*n* = 167), and low affective burden (*n* = 267). Few cross-domain edges were stable under bootstrap (five in the overall sample; six, three, and none in the profiles), and direction certainty was close to 0.50 for most. The two edges with the highest direction certainty, orientation to retardation and language to anxiety/somatization, ran from cognitive to affective nodes where their direction was determined, before and after adjustment for age and education. The profiles differed in which cross-domain edges appeared, not in the direction of any edge. Exploratory network comparison indicated differences between the high and low affective burden profiles in global strength and structure, with no edge-level difference surviving correction for multiple comparisons.

**Conclusion:**

Cognitive and affective domains formed largely separate communities linked by a narrow bridge, and the sample comprised three distinct profiles. The directional structures were exploratory. The findings support stratified assessment in cognitive screening settings; they do not establish a causal direction or a treatment sequence, which require longitudinal and interventional studies.

## 1 Introduction

Anxiety and depression frequently accompany cognitive complaints in patients referred for cognitive screening. Much of the epidemiological evidence on this co-occurrence comes from studies of mild cognitive impairment (MCI) in later life. MCI affects approximately one in five older adults worldwide, with higher prevalence in hospital-based than in community-based samples (1). A meta-analysis of 39 studies covering 10,587 patients reported a pooled anxiety prevalence of 21% among patients with MCI, rising to 31% in clinic-based samples (2), and a separate meta-analysis of 57 studies found that 32% of patients with MCI had clinically significant depression, reaching 40% in clinical settings (3). Cognitive screening clinics, however, serve a broader population than these studies describe: adults across the age range present with cognitive complaints, and lower cognitive performance and affective symptoms co-occur in patients who are far from old age. Whether the cognitive–affective interface described in older adults with MCI has the same structure across this wider clinical population has received little attention.

This comorbidity carries consequences beyond immediate symptom burden. The Lancet Commission on dementia prevention identified depression as one of 12 modifiable risk factors for dementia (4), and a meta-analysis of community-based cohort studies found that late-life depression approximately doubles the risk of both Alzheimer disease and vascular dementia (5). A recent longitudinal analysis of over 8,000 adults further showed that the relationship is bidirectional: depressive symptoms predicted subsequent cognitive decline, and cognitive decline predicted subsequent increases in depressive symptoms (6). This bidirectionality creates a fundamental clinical dilemma. If cognitive decline precedes anxiety and depression, clinicians should prioritize cognitive rehabilitation. If emotional disturbance erodes cognitive function, affective treatment should come first. Getting the direction wrong risks directing limited clinical resources toward the wrong target. Despite decades of research, the direction of this relationship remains unresolved, in part because most studies have analyzed heterogeneous patient populations as a single group.

Most previous studies have examined this relationship using regression or structural equation modeling, treating cognitive impairment and emotional disturbance as scale-level total scores. By collapsing individual domains and symptoms into aggregate scores, these methods cannot reveal which specific cognitive function connects to which specific affective symptom.

The network approach to psychopathology offers an alternative. In a network model, each symptom or cognitive domain is a node, and edges represent direct statistical associations after controlling for all other nodes (7). Centrality indices quantify each node’s structural importance, and bridge centrality identifies nodes that link otherwise separate clinical domains (8).

Several groups have applied network analysis to the cognitive-affective interface. Harlev et al. (9) used the Addenbrooke’s Cognitive Examination-Revised and the Hospital Anxiety and Depression Scale to map cognitive-affective networks across age groups, finding that the key bridge node shifted from dysphoria in younger adults to anhedonia in older adults. Zhang et al. (10) constructed a network from the MoCA-5min and the PHQ-9 in community-dwelling older adults, identifying sad mood as the primary bridge between depressive symptoms and cognitive domains. Chavez-Baldini et al. (11) built a transdiagnostic cognitive-psychopathology network in 1,016 psychiatric patients and found that cognition and psychopathology formed largely separate clusters, with depression, anxiety, verbal memory, and visual attention serving as the most central nodes.

These studies share three limitations. First, none used the full seven-domain MoCA as network nodes. Harlev et al. (9) used the ACE-R, which has limited clinical adoption compared to the MoCA. Zhang et al. (10) used the MoCA-5min, which covers only four cognitive domains. A complete seven-domain profile would capture finer distinctions among cognitive functions. Second, all three studies assessed emotional symptoms with self-report instruments. Self-report and clinician-rated scales differ in measurement precision and susceptibility to common method bias. When both the cognitive and affective measures come from the same informant, shared method variance can inflate cross-domain correlations. No study has used clinician-rated HAMA and HAMD as affective nodes in a cognitive-affective network. Third, all three studies used undirected networks only. An undirected edge between cognition and depression shows that they are associated but cannot indicate which influences which. Yet the direction of the relationship is the question that matters most for clinical decision-making, and cross-sectional data can at best generate directional hypotheses for longitudinal testing.

To address these gaps, the present study proposed a three-layer analytic framework. At the indicator level, we integrated the full seven-domain MoCA with clinician-rated HAMA and HAMD-24, yielding 16 nodes that combined objective cognitive assessment with clinician-rated affective evaluation. At the population level, we used latent profile analysis to identify data-driven subtypes, rather than treating the sample as a homogeneous group. At the analytic level, we combined Gaussian graphical models with Bayesian directed acyclic graphs, following the dual-method framework that McNally et al. (12) applied in bipolar disorder. This combination allowed us to move from identifying which nodes are associated to exploring, in a hypothesis-generating way, which cross-domain associations might be directed.

This study had four aims: (1) to map the microstructure of cognitive-affective interactions using GGM network analysis and identify cross-domain bridge nodes; (2) to identify cognitive-affective subtypes through latent profile analysis, validated externally with MMSE scores; (3) to explore the direction of associations between cognitive and affective nodes using Bayesian DAG analysis in the overall sample and within each subtype; and (4) to compare network structures across subtypes using the network comparison test.

## 2 Methods

### 2.1 Participants

This was a single-center retrospective cross-sectional study conducted at the Affiliated Nanhua Hospital of University of South China. Data were exported from the scale assessment system of the hospital’s sleep medicine center. Patients are referred to this service by their treating physicians when they report memory or other cognitive complaints. Referrals come mainly from the sleep medicine center, the department of neurology, and the department of geriatrics, with smaller numbers from other departments, and include both outpatients and inpatients; the system does not record inpatient or outpatient status. Assessments are administered by medical staff who hold the national psychotherapist certification and have received formal training in structured psychiatric rating, with psychiatrists available in the department. Patients who cannot cooperate with testing because of acute psychiatric symptoms are not assessed and do not appear in the system. The system stores scale scores and assessment dates together with age, sex, and education. Reasons for referral, psychiatric and neurological diagnoses, medical comorbidities, sleep disorders, and current medications are held in the separate hospital clinical record system, which covers outpatient and inpatient care and shares no record identifier with the assessment system. Individual assessments could not be reliably matched to clinical records or prescriptions in this retrospective study, so these variables, including whether a patient was receiving anxiolytic or antidepressant medication at the time of assessment, were not available for analysis. Adults aged 18 years or older who completed the Montreal Cognitive Assessment (MoCA), the Hamilton Anxiety Rating Scale (HAMA), and the 24-item Hamilton Depression Rating Scale (HAMD-24) within 7 days of each other were eligible for inclusion. Records with any missing item-level data were excluded. For patients with multiple assessments, only the first was retained. The final analytic sample comprised 548 patients. Participants ranged from 18 to 89 years of age (mean 58.2 ± 13.9 years), and approximately half were aged 60 years or older. No age restriction beyond adulthood was applied; the sample therefore reflects the full adult age range of the referral population.

This sample size met recommended thresholds for all planned analyses. For Gaussian graphical model estimation with 16 nodes, simulation studies have demonstrated that 250 to 350 observations provide adequate power (13). For latent profile analysis, the smallest identified profile contained 114 cases (20.8%), well above the commonly applied minimum proportion of 5% (14). For Bayesian directed acyclic graph structure learning, the sample yielded over 34 observations per node.

The study was approved by the Ethics Committee of the Affiliated Nanhua Hospital of University of South China (No. 2026-ky-013). All data were de-identified prior to analysis.

### 2.2 Measures

The 16 network nodes were derived from three clinician-administered instruments covering cognitive function and affective symptoms.

Cognitive function was assessed with the Montreal Cognitive Assessment (MoCA; 15). Seven domain scores served as cognitive nodes: visuospatial/executive function (VE, 0–5), naming (NAM, 0–3), delayed recall (MEM, 0–5), attention (ATT, 0–6), language (LANG, 0–3), abstraction (ABS, 0–2), and orientation (ORI, 0–6). One point was added to the total score for individuals with 12 years of education or less, following the standard correction procedure. An education-corrected total score below 26 was used only as a descriptive marker of low performance on the screening instrument. It was not used to diagnose mild cognitive impairment. A brief screening test applied with a single cutoff cannot establish that diagnosis without clinical history and functional assessment (16), and these data were not available in the present study.

Anxiety was assessed with the Hamilton Anxiety Rating Scale (HAMA; 17), which yields two factor scores: psychic anxiety (HA_P) and somatic anxiety (HA_S). Depression was assessed with the 24-item Hamilton Depression Rating Scale (HAMD-24; 18), which yields seven factor scores: anxiety/somatization (HD_F1), weight (HD_F2), cognitive disturbance (HD_F3), diurnal variation (HD_F4), retardation (HD_F5), sleep disturbance (HD_F6), and hopelessness (HD_F7). Both the HAMA and HAMD-24 were administered by clinicians who had received formal training in structured psychiatric rating. Unlike the self-report instruments used in prior cognitive-affective network studies, such as the Hospital Anxiety and Depression Scale (9) and the Patient Health Questionnaire-9 (10), clinician-rated scales reduce the risk of common method bias that can inflate cross-domain correlations.

The 16 nodes were grouped into two *a priori* communities for bridge centrality analysis: a cognitive community (7 MoCA domains) and an affective community (9 HAMA/HAMD-24 factors). The Mini-Mental State Examination (MMSE) total score served as an external validation variable for latent profile analysis and was not included in the network model. MMSE scores were available for 382 participants (69.7%). At the assessment service, the treating physician orders one of two assessment combinations: the MMSE with the MoCA for patients with cognitive complaints and no obvious emotional problems, or the HAMA and HAMD-24 with the MoCA for patients whose complaints include emotional or sleep problems. Participants with MMSE data were those for whom both combinations had been ordered. MMSE availability therefore reflected physician ordering practice rather than a study protocol, and participants with and without MMSE data were compared on demographic, cognitive, affective, and profile variables (Section 2.3.8).

### 2.3 Network analysis

All analyses were performed in R version 4.5.1 (19). The 16 indicators were standardized to Z-scores for the network and directed acyclic graph analyses; latent profile analysis was fitted to the original scores (Section 2.3.1). Shapiro-Wilk tests indicated significant departures from normality for all 16 nodes (all *p* < 0.001). Several MoCA domains also exhibited substantial ceiling effects: 78.6% of participants scored at maximum on naming, 64.8% on orientation, 61.9% on language, and 59.5% on abstraction. Given these distributional properties, Spearman rank correlations were used as input for all network models.

The primary R packages were tidyLPA 2.0.2 (20) for latent profile analysis, calling mclust 6.1.2 (21) internally; bootnet 1.8 (22) for network estimation and stability testing; qgraph 1.9.8 (23) for network visualization and centrality computation; networktools 1.6.0 (24) for bridge centrality; NetworkComparisonTest 2.2.3 (25) for permutation-based network comparison; and bnlearn (26) for directed acyclic graph analysis.

#### 2.3.1 Latent profile analysis

Latent profile analysis was conducted using the tidyLPA package (20), which calls mclust internally (21). Models with one to six profiles were fitted to the 16 indicators on their original scales. The covariance structure was specified as Model 1, corresponding to the EEI parameterization in mclust, which constrains variances to be equal across profiles and fixes all covariances to zero. This is the most parsimonious specification and reduces the risk of overfitting when the number of indicators is large relative to within-profile sample sizes. For the three-profile solution, we also fitted the three alternative specifications available in tidyLPA (free variances with zero covariances; equal variances with equal covariances; free variances and covariances). The two specifications with free variances could not be estimated. The equal-covariance specification had a lower BIC than Model 1 but required 306 parameters, had higher CAIC and AWE, lower entropy, and reassigned 30.3% of participants, mainly between Profiles 2 and 3 (**Supplementary Table S2**). Model 1 was retained as the most parsimonious estimable specification with the highest classification quality.

The number of profiles was chosen by considering the information criteria (AIC, BIC, and sample-size adjusted BIC), entropy of 0.80 or above, the bootstrap likelihood ratio test (100 bootstrap replications), a minimum profile proportion of 5%, average posterior classification probabilities, and clinical interpretability (14). Full fit indices for one to six profiles are given in **Supplementary Table S1**. After profile assignment, demographic and clinical characteristics were compared across profiles using one-way ANOVA or Kruskal-Wallis tests for continuous variables and chi-squared tests for categorical variables. The Mini-Mental State Examination total score was used as an external validation variable to evaluate whether the identified profiles were distinguishable on an independent cognitive measure not included in the network model.

#### 2.3.2 Network estimation

Gaussian graphical models were estimated using the EBICglasso algorithm implemented in the bootnet package (22). The input was a Spearman correlation matrix, and the extended Bayesian information criterion tuning parameter γ was set to 0.5 to balance sparsity against sensitivity. Edge weights represent regularized partial correlation coefficients. Networks were estimated separately for the overall sample and for each latent profile.

The same specification (Spearman correlations, EBICglasso, γ = 0.5) was used for the overall network and for each profile-specific network, so that networks were comparable across samples and the network comparison test could be applied without re-estimation. Because Profile 1 was the smallest profile (*n* = 114), its network was also re-estimated with γ set to 0 as a sensitivity check (Section 3.7).

#### 2.3.3 Centrality and bridge centrality

Expected influence (EI) was computed for each node to quantify its overall influence within the network (27). Unlike strength centrality, which sums absolute edge weights, EI retains the sign of each edge, making it more appropriate for networks containing both positive and negative connections. Nodes with high EI exert a strong net activating or inhibiting effect on the network as a whole.

Bridge expected influence (bEI) was computed to identify nodes that transmit activation between the cognitive and affective communities (28). The two communities were defined *a priori* based on instrument source: a cognitive community comprising the seven MoCA domains and an affective community comprising the two HAMA factors and seven HAMD-24 factors. Nodes with the highest absolute bEI values were considered core bridge nodes linking the two domains.

#### 2.3.4 Network stability

Two bootstrap procedures implemented in the bootnet package (22) were used to assess network stability. First, a case-dropping bootstrap estimated the stability of centrality orderings by progressively removing random subsets of participants up to 75% of the sample. The correlation stability coefficient (CS) was computed for both EI and bEI, defined as the maximum proportion of cases that can be dropped while retaining a correlation of at least 0.7 with the original centrality ordering at 95% certainty. A CS of 0.25 or above is considered acceptable and 0.50 or above is considered ideal. Second, a nonparametric bootstrap with 1000 resamples was used to estimate 95% confidence intervals for edge weights.

#### 2.3.5 Directed acyclic graph analysis

To explore the directionality of associations between cognitive and affective nodes, Bayesian network structure learning was performed using the bnlearn package (26), following the analytic framework applied by McNally et al. (12) in bipolar disorder symptom networks. The hill-climbing score-based algorithm was used with the Gaussian Bayesian information criterion as the scoring function. To reduce the risk of converging on local optima, 50 random restarts were performed with 100 perturbations per restart. The stability of the DAG structure was assessed with 1000 nonparametric bootstrap resamples, with the search re-run on each resample. An edge appearing in 50% or more of bootstrap resamples was considered stable, and 85% or more was considered highly stable. For stable edges, the direction observed in more than 50% of bootstrap resamples was reported, with the proportion serving as an index of direction certainty. Both measures describe how often an edge and its direction were reproduced under resampling; they do not indicate whether the edge or its direction is correct.

DAGs were estimated separately for the overall sample and for each latent profile using the same algorithm and parameters. For each DAG, the completed partially directed acyclic graph was obtained (bnlearn cpdag), and each cross-domain edge was classified as compelled, meaning that its direction is determined by v-structures and is the same for every DAG in the Markov equivalence class, or reversible. Because the Gaussian BIC is score-equivalent, all DAGs in the same equivalence class receive the same score, and the direction of a reversible edge reflects the search path rather than the data; directions were therefore interpreted only for compelled edges, and only as exploratory. Cross-domain edges were then compared between the overall and profile-specific DAGs edge by edge, in terms of bootstrap inclusion and direction certainty, to examine whether the same edges appeared with the same or opposite direction across samples or whether different edges appeared.

#### 2.3.6 Network comparison test

Pairwise comparisons between latent profile networks were conducted using the NetworkComparisonTest package (25) with 1000 permutations per comparison.

Three levels of invariance were tested. Global strength invariance tested whether the sum of absolute edge weights differed between the two networks. Network structure invariance tested whether the largest individual edge weight difference exceeded what would be expected by chance. Edge-level invariance tested each edge individually for weight differences between the two networks. All tests used a significance threshold of 0.05. Edge-level testing was performed only for profile pairs that showed differences on both global strength and network structure invariance, to limit the number of tests. No correction for multiple comparisons was applied, and the results of the network comparison test are reported as exploratory.

#### 2.3.7 Sensitivity analyses

Five sensitivity analyses were conducted to assess the robustness of the main findings.

First, a 14-node model was estimated after removing naming and abstraction, the two MoCA domains with the narrowest score ranges and pronounced ceiling effects. As an additional check, a second 14-node model was estimated after removing naming and orientation, the two domains with the highest ceiling rates. Expected influence rankings from these reduced models were compared with those from the full 16-node model.

Second, a 15-node model was estimated by combining naming and language into a single composite node, given their shared linguistic basis. Expected influence rankings were again compared with the full model.

Third, the overall network was re-estimated with the tuning parameter γ set to 0, which removes the sparsity penalty entirely and retains all edges supported by the data. The top three nodes by expected influence were compared with those from the primary model to assess whether centrality rankings depended on the degree of regularization.

Fourth, data-driven community detection was performed using both the walktrap and spinglass algorithms. The resulting community assignments were compared with the *a priori* two-community partition using normalized mutual information (NMI), where a value of 1.0 indicates perfect agreement. This analysis tested whether the theoretical division into cognitive and affective communities was supported by the empirical network structure.

Fifth, the overall and profile-specific DAGs were re-estimated using the tabu search algorithm in place of hill-climbing, with the same scoring function. For each sample, the two point-estimate graphs were compared by the structural Hamming distance and, for cross-domain edges, edge by edge, classifying each edge as identical in both graphs, present in both with opposite direction, or present in one graph only. This assessed whether the cross-domain edges and their directions were sensitive to the choice of structure learning algorithm. Tabu search was not bootstrapped; bootstrap values cited in the comparison refer to the hill-climbing analysis.

#### 2.3.8 Sensitivity analyses for age, education, and age subgroup

Three additional analyses examined whether age and education could account for the profiles or the network findings. First, a multinomial logistic regression (nnet package) modelled profile membership as a function of age (years) and education (ordinal, six levels: illiterate, primary school, junior middle school, senior middle school, college or university, and graduate), with Profile 1 as the reference. Explanatory power was summarized by McFadden’s pseudo *R*² and by the proportion of participants correctly classified, compared with the proportion obtained by assigning all participants to the largest profile. Second, each of the 16 indicators was standardized and regressed on age and education, and the residuals were used to re-estimate the overall network with the same EBICglasso procedure, followed by expected influence, bridge expected influence, community detection, and case-dropping bootstrap. Directed acyclic graphs were re-estimated on the residualized data for the overall sample and for each profile, using hill-climbing with 500 bootstrap replications. Participants with missing education (*n* = 5) were excluded from these analyses. Third, the overall network was re-estimated in the subgroup aged 60 years or older, using the same procedures as the main analysis. In addition, participants with and without MMSE data were compared on age, education, sex, MoCA total score, HAMA and HAMD-24 total scores, and profile membership, using Welch t tests for continuous variables and chi-square tests for categorical variables.

## 3 Results

### 3.1 Participant characteristics

The final sample included 548 patients (309 female, 56.4%) with a mean age of 58.23 ± 13.93 years (range 18–89). Of the 543 participants with education data, 323 (59.5%) had completed junior middle school or less. In the full sample, 294 patients (53.6%) scored below the conventional cutoff of 26 on the education-corrected MoCA total. Participants with MMSE data (*n* = 382) and without (*n* = 166) did not differ in age, education, sex, MoCA total score, or profile membership (all *p* ≥ 0.287); those with MMSE data had slightly higher HAMA and HAMD-24 scores (differences of 1.6 and 1.7 points; both *p* = 0.021) (**Supplementary Table S7**). Mean scores were 22.86 ± 7.00 for the MoCA (education-corrected), 14.28 ± 7.66 for the HAMA, and 15.74 ± 7.88 for the HAMD-24.

All 16 nodes departed significantly from normality (all Shapiro-Wilk *p* < 0.001), confirming the use of Spearman correlations for all subsequent network analyses. At the correlation matrix level, the mean absolute within-domain correlation was 0.495 for cognitive nodes and 0.359 for affective nodes, whereas the mean absolute cross-domain correlation was only 0.058. This pattern indicated that cognitive and affective domains were already largely separated before any network modeling was applied (**Table 1**).

**Table 1:** Demographic, clinical, and node-level characteristics of the study sample (*N* = 548).

Characteristic			Value	
Demographic characteristics				
Age, years, mean ± SD (range)			58.23 ± 13.93 (18–89)	
Female, n (%)			309 (56.4)	
Education, n (%)				
Illiterate			33 (6.1)	
Primary school			120 (22.1)	
Junior middle school			170 (31.3)	
Senior middle school			132 (24.3)	
College or university			82 (15.1)	
Graduate or above			6 (1.1)	
Missing, n			5	
MoCA corrected total < 26, n (%)			294 (53.6)	
Clinical scale totals, mean ± SD				
MoCA (education-corrected total)			22.86 ± 7.00	
HAMA total			14.28 ± 7.66	
HAMD-24 total			15.74 ± 7.88	
Node	Full name	Mean ± SD	Range	Ceiling (%)
Cognitive community (MoCA)				
VE	Visuospatial/executive	3.53 ± 1.63	0–5	39.8
NAM	Naming	2.66 ± 0.75	0–3	78.6
MEM	Delayed recall	2.21 ± 1.86	0–5	16.6
ATT	Attention	4.73 ± 1.66	0–6	47.4
LANG	Language	2.31 ± 1.02	0–3	61.9
ABS	Abstraction	1.39 ± 0.81	0–2	59.5
ORI	Orientation	5.24 ± 1.40	0–6	64.8
Affective community				
HA_P	Psychic anxiety	8.54 ± 4.48	0–25	—
HA_S	Somatic anxiety	5.73 ± 3.92	0–25	—
HD_F1	Anxiety/somatization	4.61 ± 2.26	0–14	—
HD_F2	Weight	0.33 ± 0.63	0–2	—
HD_F3	Cognitive disturbance	3.09 ± 2.43	0–15	—
HD_F4	Diurnal variation	0.46 ± 0.59	0–2	—
HD_F5	Retardation	2.46 ± 2.03	0–12	—
HD_F6	Sleep disturbance	3.26 ± 1.76	0–6	—
HD_F7	Hopelessness	1.53 ± 1.76	0–11	—

MoCA, Montreal Cognitive Assessment; HAMA, Hamilton Anxiety Rating Scale; HAMD-24, 24-item Hamilton Depression Rating Scale. Education percentages are based on the 543 participants with available data (5 missing). Cognitive community nodes are MoCA domain scores; affective community nodes comprise two HAMA factors and seven HAMD-24 factors. Ceiling (%) = percentage of participants scoring at the domain maximum. Affective factor scores have wide ranges and ceiling effects were not a primary concern (— indicates not applicable). All 16 nodes departed significantly from normality (Shapiro-Wilk *p* < 0.001 for all); Spearman correlations were used throughout.

### 3.2 Latent profile analysis identified three qualitatively distinct profiles

Models with one to six profiles were fitted to the 16 indicators (**Supplementary Table S1**). AIC, BIC, and sample-size adjusted BIC all continued to decrease up to six profiles and did not reach a minimum, and the bootstrap likelihood ratio test was significant at every step, so neither criterion identified a single solution. The largest decrease in BIC occurred from two to three profiles (1401 points); subsequent decreases were smaller (268, 513, and 187 points). From four profiles onward, the smallest profile contained fewer than 5% of the sample (22, 25, and 21 participants). The three-profile solution had an entropy of 0.891, average posterior classification probabilities of 0.965, 0.929, and 0.958 for the three profiles, and a smallest profile of 20.8%. We retained the three-profile solution because it was the largest number of profiles that satisfied the minimum profile size criterion, its classification quality was high, and its profiles were clinically interpretable. We note that the information criteria alone would have favored a larger number of profiles.

The three profiles did not form a severity continuum but represented qualitatively distinct clinical presentations (**Figure 1**; **Table 2**). Profile labels describe the feature that distinguished each profile within this sample. They are relative descriptions and do not indicate diagnostic status.

**Figure 1:**
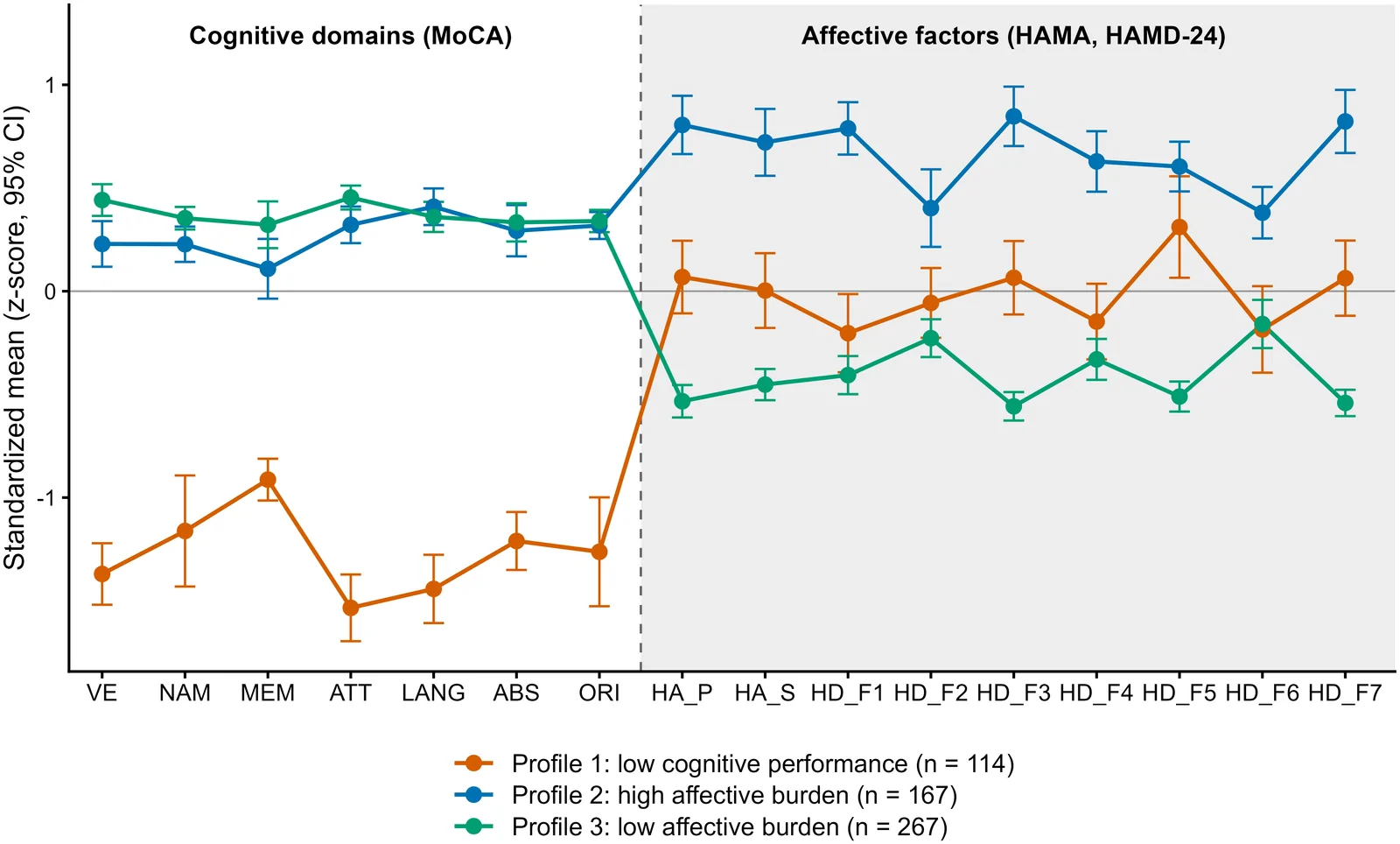
Standardized indicator means for the three latent profiles. Points are mean z-scores of each of the 16 indicators (standardized across the full sample, *n* = 548) and bars are 95% confidence intervals. Latent profile analysis was fitted on raw scores; values are standardized here for display only. The shaded area marks the affective indicators. Profile 1, low cognitive performance (*n* = 114); Profile 2, high affective burden (*n* = 167); Profile 3, low affective burden (*n* = 267). VE, visuospatial/executive; NAM, naming; MEM, delayed recall; ATT, attention; LANG, language; ABS, abstraction; ORI, orientation; HA_P, psychic anxiety; HA_S, somatic anxiety; HD_F1, anxiety/somatization; HD_F2, weight; HD_F3, cognitive disturbance; HD_F4, diurnal variation; HD_F5, retardation; HD_F6, sleep disturbance; HD_F7, hopelessness.

**Table 2:** Comparison of demographic and clinical characteristics across the three latent profiles.

Variable	Profile 1 (n = 114)	Profile 2 (n = 167)	Profile 3 (n = 267)	p
Demographics				
Age, years	68.0 ± 10.1	55.8 ± 13.5	55.6 ± 13.8	<0.001
Female, n (%)	79 (69.3)	96 (57.5)	134 (50.2)	0.003
Education, n (%)				<0.001
Illiterate	25 (22.1)	3 (1.8)	5 (1.9)	
Primary school	45 (39.8)	31 (18.7)	44 (16.7)	
Junior middle school	25 (22.1)	55 (33.1)	90 (34.1)	
Senior middle school	17 (15.0)	43 (25.9)	72 (27.3)	
College or above	1 (0.9)	34 (20.5)	53 (20.1)	
Clinical measures				
MoCA total	10.5 ± 4.9	24.4 ± 3.8	25.5 ± 3.7	<0.001
MoCA corrected total	11.5 ± 4.9	25.2 ± 3.7	26.3 ± 3.5	<0.001
MoCA corrected total < 26, n (%)	114 (100)	81 (48.5)	99 (37.1)	<0.001
HAMA total	14.6 ± 7.5	20.7 ± 7.4	10.1 ± 4.4	<0.001
HAMD-24 total	15.7 ± 7.9	23.6 ± 5.8	10.9 ± 4.3	<0.001
External validation				
MMSE subset, n	81	123	178	
MMSE total	17.2 ± 7.1	27.1 ± 2.8	28.0 ± 2.3	<0.001

Values are mean ± SD or n (%). MoCA, Montreal Cognitive Assessment; HAMA, Hamilton Anxiety Rating Scale; HAMD-24, 24-item Hamilton Depression Rating Scale; MMSE, Mini-Mental State Examination. Education percentages are based on participants with available data (5 missing across all profiles). College or above combines college/university and graduate education. Between-group comparisons used one-way ANOVA or Kruskal-Wallis tests for continuous variables and chi-squared tests for categorical variables. MMSE data were available for a subset of participants and served as external validation; Tukey post-hoc tests showed *p* < 0.001 for Profile 1 vs. Profiles 2 and 3, and *p* = 0.160 for Profile 2 vs. Profile 3. Profile 1, low cognitive performance; Profile 2, high affective burden; Profile 3, low affective burden.

Profile 1 (low cognitive performance; *n* = 114, 20.8%) had the lowest cognitive scores in the sample. The mean MoCA score was 10.5 ± 4.9, and all 114 patients scored below the cutoff. This group was substantially older than the other two (68.0 ± 10.1 years vs. approximately 56 years in Profiles 2 and 3). Affective symptoms were close to the overall sample mean (HAMA 14.6 ± 7.5; HAMD-24 15.7 ± 7.9).

Profile 2 (high affective burden; *n* = 167, 30.5%) had a mean MoCA of 24.4 ± 3.8, with 48.5% scoring below the cutoff, and carried the highest affective burden of any group (HAMA 20.7 ± 7.4; HAMD-24 23.6 ± 5.8). The mean age was 55.8 ± 13.5 years.

Profile 3 (low affective burden; *n* = 267, 48.7%) had cognitive scores comparable to Profile 2 (MoCA 25.5 ± 3.7; 37.1% below the cutoff) but the lowest affective scores (HAMA 10.1 ± 4.4; HAMD-24 10.9 ± 4.3). The mean age was 55.6 ± 13.8 years. All between-profile comparisons on age, education, and scale totals were statistically significant (all *p* < 0.001; sex, *p* = 0.003).

MMSE data were available for 382 of the 548 participants (69.7%) and served as external validation. Profile 1 had a mean MMSE of 17.2 ± 7.1, substantially lower than Profile 2 (27.1 ± 2.8; *p* < 0.001, *d* = 1.84) and Profile 3 (28.0 ± 2.3; *p* < 0.001, *d* = 2.05). Profiles 2 and 3 did not differ on MMSE (*p* = 0.160), consistent with these two groups being distinguished by affective symptom burden rather than cognitive level. Effect sizes (*d*) were calculated with the root mean square of the two group standard deviations as the standardizer.

### 3.3 Overall network structure: two separated cognitive–affective communities

The 16-node GGM network displayed a pronounced two-community structure (**Figure 2**). Both the walktrap and spinglass community detection algorithms perfectly recovered the *a priori* partition into cognitive and affective communities (NMI = 1.000 for both), with all 16 nodes correctly assigned to their respective domain. Of the edges retained after EBICglasso regularization, only 13 crossed the domain boundary. The strongest cross-domain edge, between LANG and HD_F1, had a regularized partial correlation of only 0.078. This degree of separation was more extreme than the cognitive–affective community structure reported by Harlev et al. (9) using ACE-R and HADS, where cross-domain connectivity was substantially higher.

**Figure 2:**
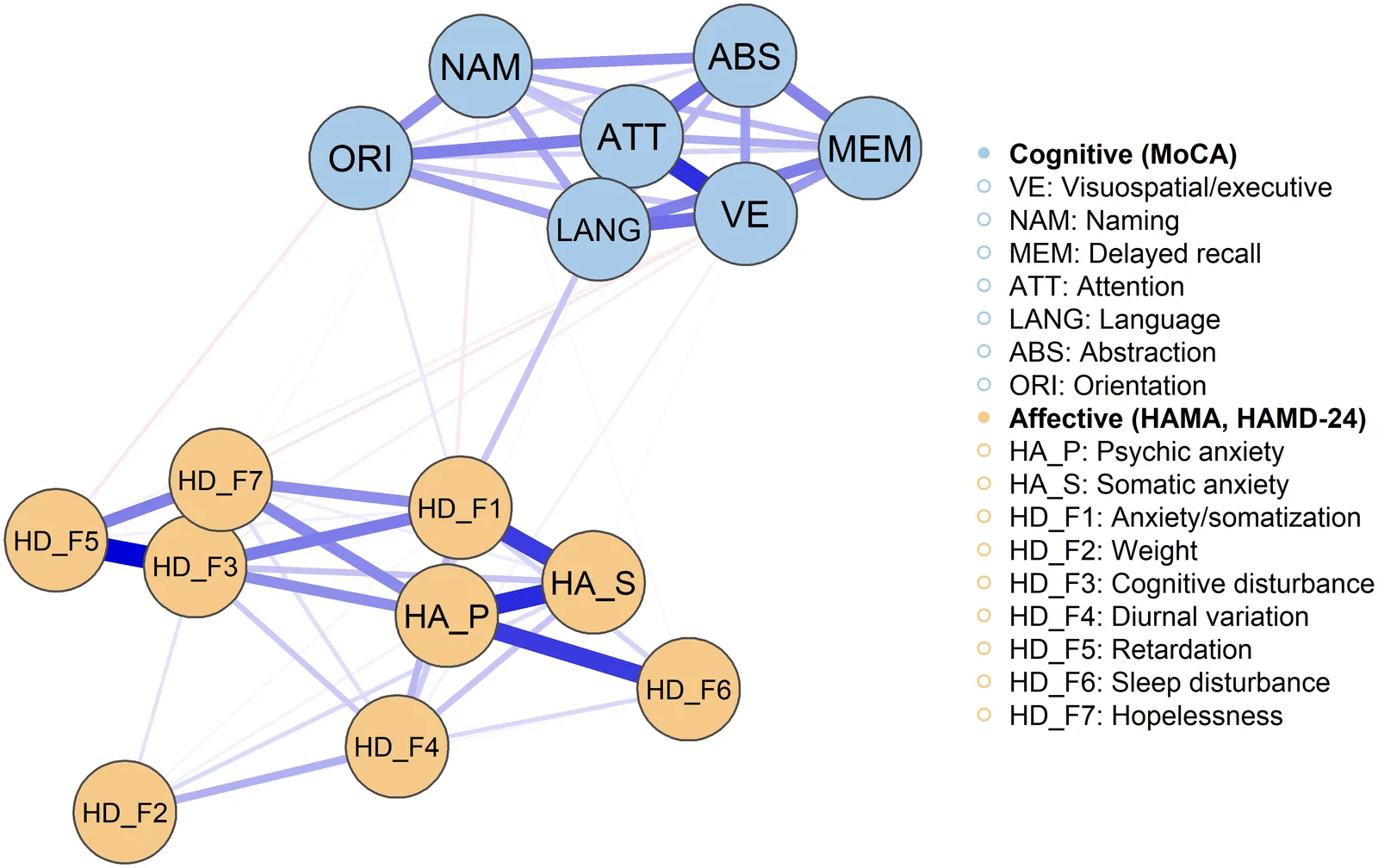
Gaussian graphical model of the 16-node cognitive-affective network in the overall sample (*n* = 548), estimated with EBICglasso (γ = 0.5) on Spearman correlations. Edges are regularized partial correlations; edge width is proportional to absolute weight, blue edges are positive and red edges negative. Blue nodes are MoCA cognitive domains and orange nodes are HAMA and HAMD-24 affective factors. Node positions were determined by the Fruchterman-Reingold algorithm and are kept identical in **Figures 3** and **4**. Abbreviations as in **Figure 1**.

### 3.4 Centrality and bridge symptoms

Among the 16 nodes, HA_P (psychic anxiety, EI = 1.127) and HD_F3 (cognitive disturbance, EI = 1.036) had the highest expected influence, followed by ATT (attention, EI = 1.006) and HD_F1 (anxiety/somatization, EI = 0.975). Within the cognitive community, ATT and LANG (language, EI = 0.962) ranked first and second. Within the affective community, the top two nodes were HA_P and HD_F3 (**Table 3**).

**Table 3:** Expected influence (EI) and bridge expected influence (bEI) for 16 network nodes.

Node	Full name	Community	EI	EI rank	bEI
Affective community					
HA_P	Psychic anxiety	Affective	1.127	1	-0.029
HD_F3	Cognitive disturbance	Affective	1.036	2	-0.034
HD_F1	Anxiety/somatization	Affective	0.975	4	+0.109
HA_S	Somatic anxiety	Affective	0.792	8	-0.008
HD_F7	Hopelessness	Affective	0.751	10	-0.037
HD_F5	Retardation	Affective	0.654	11	-0.051
HD_F4	Diurnal variation	Affective	0.551	14	+0.026
HD_F6	Sleep disturbance	Affective	0.406	15	+0.010
HD_F2	Weight	Affective	0.245	16	0
Cognitive community					
ATT	Attention	Cognitive	1.006	3	0
LANG	Language	Cognitive	0.962	5	+0.086
VE	Visuospatial/executive	Cognitive	0.852	6	-0.064
ABS	Abstraction	Cognitive	0.794	7	0
MEM	Delayed recall	Cognitive	0.754	9	0
ORI	Orientation	Cognitive	0.647	12	+0.001
NAM	Naming	Cognitive	0.645	13	-0.037

EI, expected influence; bEI, bridge expected influence. Nodes are grouped by community and sorted by EI within each community. EI rank is the overall rank across all 16 nodes. Positive bEI indicates net activating cross-domain influence; negative bEI indicates net inhibitory cross-domain influence; zero indicates no cross-domain edges. Case-dropping bootstrap stability: CS(EI) = 0.750 (ideal, above 0.50 threshold); CS(bEI) = 0.361 (acceptable, above 0.25 threshold).

Bridge expected influence identified two primary cross-domain bridge nodes: HD_F1 (bEI = +0.109) on the affective side and LANG (bEI = +0.086) on the cognitive side. These two nodes were directly connected by the strongest cross-domain edge in the network (regularized partial correlation = 0.078). The third-ranked bridge node was VE (bEI = -0.064), whose negative value indicated that its cross-domain connections were predominantly inhibitory. Four nodes had a bEI of exactly zero, meaning they did not participate in any cross-domain edge: MEM, ATT, ABS, and HD_F2.

Case-dropping bootstrap analysis indicated that the EI ordering was highly stable (CS = 0.750, exceeding the ideal threshold of 0.50), while the bEI ordering met the acceptable threshold (CS = 0.361, above 0.25) (**Table 3**).

### 3.5 Profile-specific networks and network comparison

The three profile-specific networks displayed markedly different topologies (**Figure 3**).

**Figure 3:**
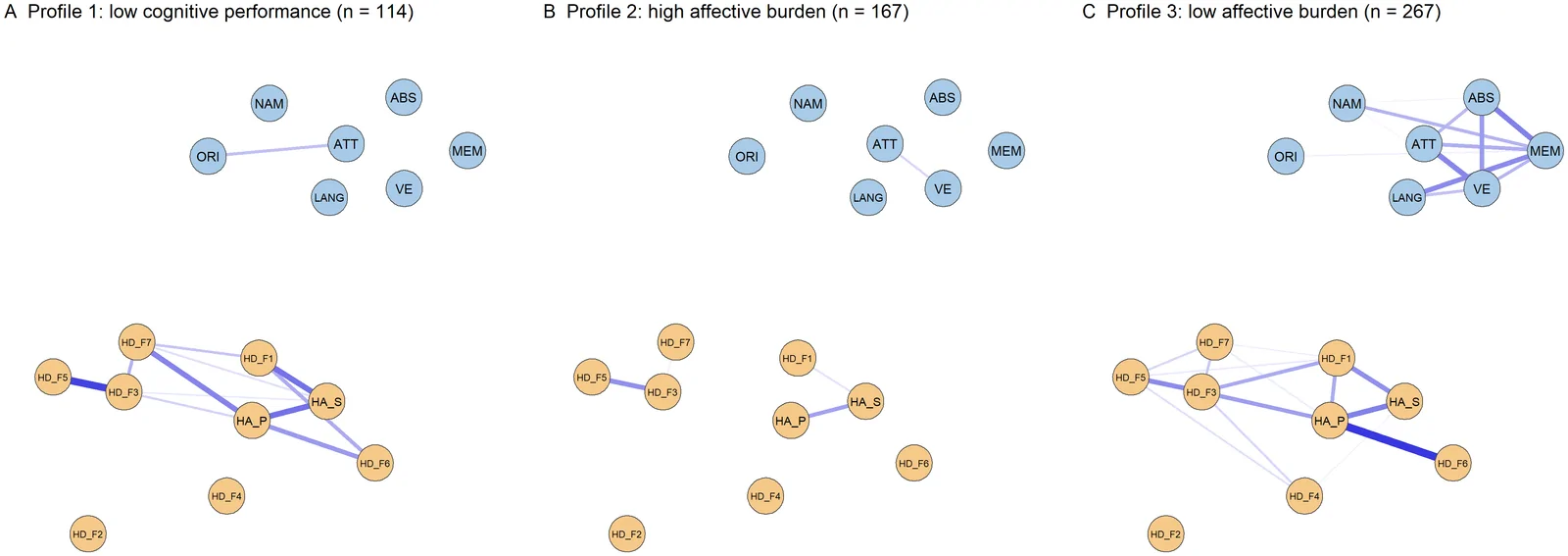
Profile-specific Gaussian graphical models for Profile 1 [**(A)**, *n* = 114], Profile 2 [**(B)**, *n* = 167], and Profile 3 [**(C)**, *n* = 267], estimated with the same specification as **Figure 2** (EBICglasso, γ = 0.5, Spearman correlations). Node layout and edge-width scale are identical to **Figure 2**, so edge widths are comparable across panels. Abbreviations as in **Figure 1**.

In Profile 1, five of the seven cognitive nodes had an EI of zero, and the remaining two (ATT and ORI) retained only minimal connectivity (EI = 0.085 each). The network was dominated by affective nodes, with HA_P ranking first (EI = 0.562). This pattern likely reflected the combination of floor effects across multiple MoCA domains in this group with very low MoCA scores (mean = 10.5) and the tendency of L1 regularization to shrink low-variance partial correlations to zero.

Profile 2 presented the sparsest network of the three. HD_F3 replaced HA_P as the highest-EI node (0.176), and most nodes had no connections at all. Profile 3, by contrast, had the densest network, with 15 of 16 nodes showing nonzero EI. HA_P returned to the top position (EI = 0.827), and MEM rose to second (EI = 0.677), a pattern unique to this profile.

Profiles 2 and 3 had comparable cognitive scores (MoCA 24.4 vs. 25.5), so if ceiling effects alone explained network sparsity, the two networks should have looked similar. The network comparison test indicated differences between them in global strength (GSI *p* = 0.034) and network structure (NI *p* = 0.014). These are two of three pairwise comparisons and were not corrected for multiple comparisons, so they should be read as exploratory. Comparisons involving Profile 1 did not reach significance (all *p* > 0.20); given the size of this profile (*n* = 114) and the sparsity of its network, these comparisons were underpowered and do not show that Profile 1 resembled the other two profiles.

Edge-level testing of the 120 possible edges between Profiles 2 and 3 found two edges with uncorrected *p* < 0.05, both within the affective community: HA_P and HD_F6 (*p* = 0.003) and HA_P and HA_S (*p* = 0.037). Neither remained significant after Bonferroni correction (threshold 0.05/120 = 0.0004), so no individual edge can be said to differ between the two profiles. No cross-domain edge reached the uncorrected threshold (**Table 4**).

**Table 4:** Network comparison test results for pairwise profile comparisons.

Panel A. Global invariance tests				
Comparison	GSI diff	GSI p	NI diff	NI p
P1 vs. P2	1.069	0.238	0.168	0.969
P1 vs. P3	1.292	0.415	0.173	0.875
P2 vs. P3	2.361	0.034*	0.275	0.014*
Panel B. Edge-level differences with uncorrected p < 0.05 (P2 vs. P3)				
Edge	Test statistic	p		Domain
HA_P – HD_F6	0.303	0.003*		Affective
HA_P – HA_S	0.175	0.037*		Affective

GSI, global strength invariance; NI, network structure invariance. All tests used 1000 permutations. *p < 0.05, uncorrected for multiple comparisons. The three global comparisons were not corrected and are reported as exploratory. Edge-level testing was performed only for the P2 vs. P3 comparison, which showed differences on both global strength and network structure invariance. Of 120 edges tested, 2 had uncorrected *p* < 0.05, both within the affective community; neither remained significant after Bonferroni correction (threshold 0.05/120 = 0.0004). No cross-domain edge reached the uncorrected threshold. Profile 1, low cognitive performance; Profile 2, high affective burden; Profile 3, low affective burden.

### 3.6 Directed acyclic graphs: exploratory directional structures

The DAG estimated from the full sample contained 39 directed edges, 7 of which connected a cognitive and an affective node. Six of the seven cross-domain edges were compelled in the completed partially directed acyclic graph, so their direction was determined by v-structures rather than by the search order; the edge between ORI and HD_F6 was reversible. Bootstrap inclusion and direction certainty for every cross-domain edge are given in **Supplementary Table S3**. Five cross-domain edges reached the inclusion threshold of 0.50. Only one had high direction certainty: ORI to HD_F5 (inclusion 0.998, direction 0.80). The edge between ORI and HD_F6 was reversible in the point-estimate graph, so its bootstrap direction (0.584, 0.71 towards HD_F6) is reported without interpretation. LANG to HD_F1 (0.687, 0.58), VE to HD_F4 (0.683, 0.58), and ORI to HD_F1 (0.664, 0.52) had direction certainty close to 0.50, so their direction should be regarded as undetermined; for VE and HD_F4 the point-estimate DAG contained the opposite direction. Three edges in the point-estimate DAG (ORI to HA_S, HD_F1 to VE, NAM to HA_P) appeared in fewer than half of the bootstrap resamples.

The profile-specific DAGs are shown in **Figure 4** and **Supplementary Table S3**. In Profile 1 (*n* = 114), the point-estimate DAG contained six cross-domain edges, all compelled, and six cross-domain edges reached bootstrap inclusion of 0.50, with direction certainty between 0.51 and 0.64 (NAM to HA_P 0.840, 0.56, with the point-estimate direction opposite to the bootstrap majority; ORI to HD_F5 0.830, 0.64; ORI to HD_F6 0.577, 0.61; HD_F1 to ORI 0.538, 0.51; ATT to HD_F5 0.528, 0.58; VE to HA_P 0.500, 0.63).

**Figure 4:**
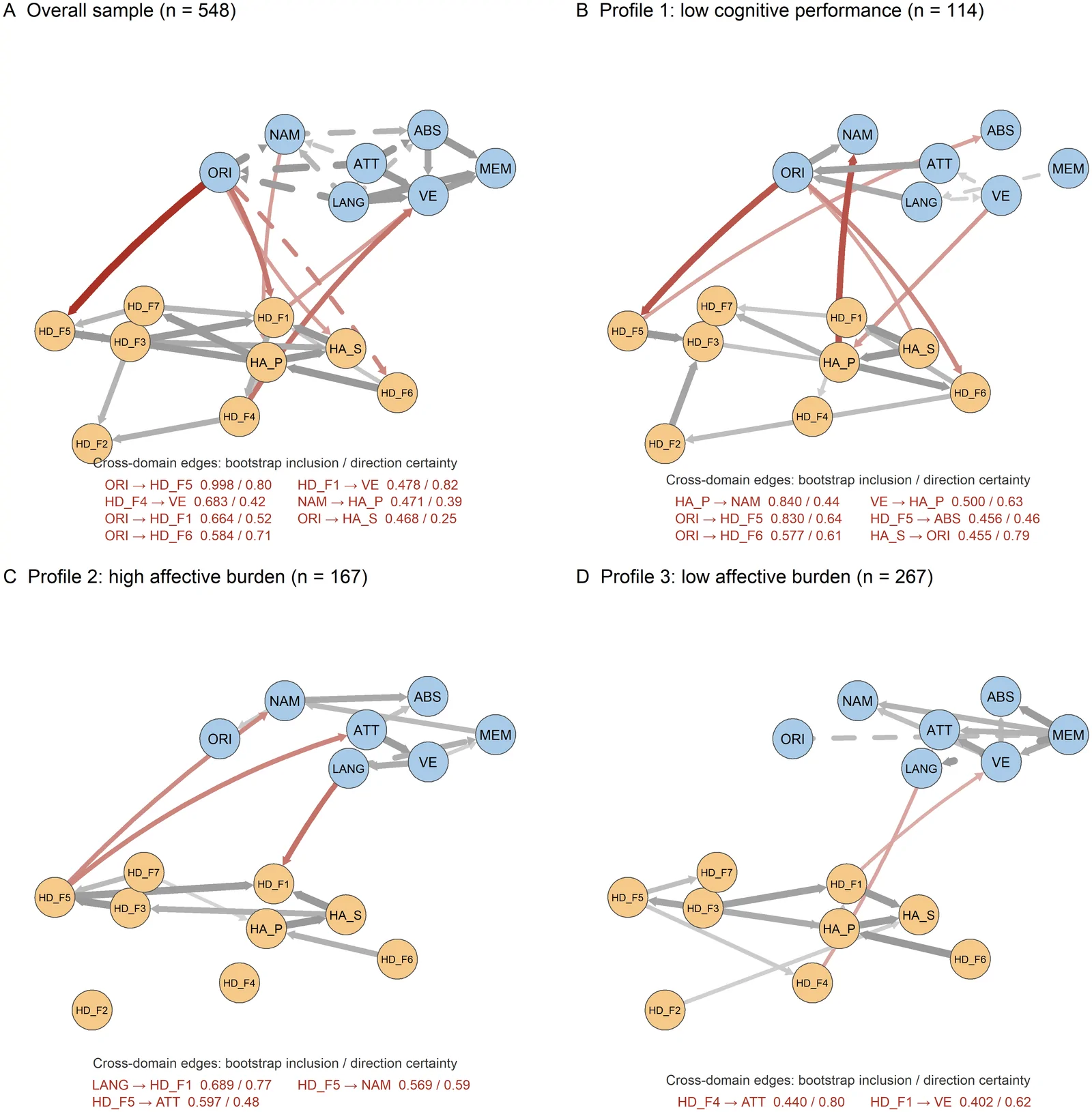
Exploratory directed acyclic graphs (DAGs) for the overall sample [**(A)**, *n* = 548] and the three profiles **(B–D)**, learned by hill-climbing with the BIC score on the unadjusted data. Arrows show the edges of the point-estimate DAG; arrow width is proportional to the proportion of 1,000 bootstrap networks containing the edge (bootstrap inclusion). Dark red arrows are cross-domain edges linking a cognitive and an affective node; grey arrows are within-domain edges. Dashed arrows were reversible in the completed partially directed acyclic graph, meaning that their direction was not fixed by the equivalence class. The list in each panel gives, for every cross-domain edge in the point-estimate DAG, its bootstrap inclusion and its direction certainty, the proportion of bootstrap networks containing the edge in which it ran in the direction drawn; values below 0.50 indicate that the majority bootstrap direction was opposite to the drawn direction. Complete values are given in **Supplementary Table S3**; results after adjustment for age and education are in **Supplementary Table S5**. Node layout as in **Figure 2**; abbreviations as in **Figure 1**.

In Profile 2 (*n* = 167), the point-estimate DAG contained three cross-domain edges, all compelled, and three reached inclusion of 0.50: LANG to HD_F1 (0.689, 0.77), ATT to HD_F5 (0.597, 0.52, with the point-estimate direction opposite to the bootstrap majority), and HD_F5 to NAM (0.569, 0.59). In Profile 3 (*n* = 267), the point-estimate DAG contained two cross-domain edges (HD_F1 to VE, HD_F4 to ATT), both compelled, but neither reached inclusion of 0.50, so no cross-domain edge was stable in this profile.

Compared edge by edge, the samples differed in which cross-domain edges appeared and how many, not in the direction of any given edge. No cross-domain edge was stable in two samples with opposite directions. The two edges with the highest direction certainty, ORI to HD_F5 and LANG to HD_F1, ran from cognitive to affective nodes wherever they appeared in the unadjusted analyses. Results after adjustment for age and education are reported in Section 3.8.

The presence of cross-domain edges in the Profile 1 DAG contrasts with the near-complete isolation of cognitive nodes in the corresponding GGM. This reflects a difference in method sensitivity: L1 regularization in the GGM shrinks weak partial correlations to zero, whereas the BIC-based score in DAG estimation retains weak conditional dependencies. Given the sample size and floor effects in Profile 1, its DAG results should be regarded as hypothesis-generating.

### 3.7 Sensitivity analyses

Results were broadly consistent across alternative model specifications. In the 14-node model excluding NAM and ABS, the three nodes with the highest EI were HA_P, HD_F3, and HD_F1, compared with HA_P, HD_F3, and ATT in the primary 16-node model, in which HD_F1 ranked fourth. The additional 14-node model excluding NAM and ORI, the two domains with the highest ceiling rates (78.6% and 64.8%), gave the same three nodes. In the 15-node model, in which NAM and LANG were combined into a single composite node, HA_P and HD_F3 remained the two nodes with the highest EI, followed by the composite node. Under a relaxed regularization parameter (gamma = 0), the top three EI nodes were identical to those under gamma = 0.5 (HA_P, HD_F3, ATT). Community detection with walktrap and spinglass algorithms both yielded perfect two-community recovery (NMI = 1.000) across all model specifications. With γ set to 0, the Profile 1 network contained 50 edges, including 18 cross-domain edges with absolute weights between 0.009 and 0.142, 11 of them negative, and the highest-EI nodes were HD_F1, HA_S, and ORI. Because this setting removes the sparsity penalty in the smallest sample and produced a dense graph of small edges, it was used only as a sensitivity check and not for inference.

For the DAG analysis, tabu search and hill-climbing were compared edge by edge. In the full sample, both algorithms returned 39 edges, but the graphs differed in 21 edges, and tabu search returned eight cross-domain edges compared with seven. Four cross-domain edges were identical in both graphs (ORI to HD_F5, ORI to HD_F6, HD_F1 to VE, HD_F4 to VE), including the edge with the highest bootstrap inclusion and direction certainty. Two node pairs were connected in both graphs with opposite directions: ORI and HD_F1, for which bootstrap direction certainty was 0.52, and NAM and HA_P, for which the tabu direction (HA_P to NAM) was the majority bootstrap direction (0.61). Three edges appeared in one graph only: ORI to HA_S under hill-climbing (bootstrap inclusion 0.468), and LANG to HD_F1 (0.687) and ORI to HD_F3 (0.351) under tabu search. In Profile 1, the graphs differed in 23 edges; four cross-domain edges were identical, none had opposite directions, two appeared only under hill-climbing (HA_S to ORI, VE to HA_P; inclusion 0.455 and 0.500), and five appeared only under tabu search, each with inclusion of 0.54 or lower. In Profiles 2 and 3, the cross-domain edges were identical under the two algorithms (three and two edges, same directions), although the full graphs differed in 10 and 5 edges. Where the algorithms disagreed, the edge concerned had bootstrap inclusion below 0.70 or direction certainty close to 0.50; where bootstrap support was strong, the algorithms agreed. The choice of algorithm therefore affected the point-estimate graphs and the direction of several cross-domain edges, which is a further reason not to interpret counts of edges in either direction.

In the 14-node and 15-node models, the bridge function could not compute bEI because the reduced node sets left too few cross-domain edges to meet the minimum computational requirements. This limitation did not affect the primary 16-node model, where the CS coefficient for bEI (0.361) exceeded the acceptable threshold of 0.25. Nonetheless, the sensitivity of bEI to node configuration is acknowledged as a limitation of this study.

### 3.8 Sensitivity analyses for age, education, and age subgroup

Age and education explained a modest part of profile membership. The multinomial model gave a McFadden pseudo *R*² of 0.129 and classified 54.5% of participants correctly, compared with 48.7% for the baseline. The odds ratios for Profile 2 and Profile 3 relative to Profile 1 were almost identical (age, 0.921 and 0.920 per year; education, 2.358 and 2.385 per level), indicating that age and education distinguished Profile 1 from the other two profiles but did not distinguish Profile 2 from Profile 3.

After residualizing the indicators on age and education (*n* = 543), no node crossed between the cognitive and affective communities; HD_F2 and HD_F4 formed a separate small community within the affective side (walktrap and spinglass NMI = 0.821). HD_F3 and HA_P remained the two nodes with the highest expected influence (1.117 and 1.063), with LANG third (1.004); the CS-coefficient for expected influence was 0.75. Bridge expected influence was more sensitive to adjustment: HD_F1 (0.101) and LANG (0.105) remained among the top three, but HD_F4 rose to the highest value (0.159); the CS-coefficient for bridge expected influence was 0.28. The adjusted network contained 19 cross-domain edges (13 in the unadjusted network), with the strongest cross-domain edge of similar magnitude (0.080 versus 0.078); the EBIC selected a low penalty for the adjusted model, so its weaker edges should be interpreted with caution (**Supplementary Table S4**).

In the adjusted DAGs (**Supplementary Table S5**), the overall sample contained nine cross-domain edges, eight of which were compelled. The edges from ORI to HD_F5 and from LANG to HD_F1 remained present and in the same direction as before adjustment (bootstrap inclusion 0.996 and 0.766; direction certainty 0.81 and 0.65), and the edge from VE to HD_F4 became more stable (inclusion 0.824; direction certainty 0.79). One edge ran from the affective to the cognitive community (HA_S to ORI; inclusion 0.510, direction certainty 0.68). In the profile-specific adjusted DAGs, ORI to HD_F5 remained in Profile 1 (inclusion 0.886; direction certainty 0.65) and LANG to HD_F1 remained in Profile 2 (0.556; 0.70). In Profile 3, an edge between HD_F1 and LANG reached inclusion (0.540) with HD_F1 to LANG as the majority direction, but its direction certainty was 0.55, so its direction is undetermined.

In the subgroup aged 60 years or older (*n* = 275; **Supplementary Table S6**), the two-community structure was preserved (walktrap NMI = 1.000; spinglass NMI = 0.875), and HD_F3 and HA_P remained the two nodes with the highest expected influence (CS-coefficient 0.593). HD_F1 remained the affective node with the highest bridge expected influence, but LANG ranked seventh, and the CS-coefficient for bridge expected influence was 0.051, below the threshold for interpretation. The subgroup network contained eight cross-domain edges, the strongest being ORI to HD_F1 (0.050).

## 4 Discussion

This study integrated seven MoCA cognitive domains with nine clinician-rated affective factors (HAMA and HAMD-24) into a unified 16-node network in 548 Chinese general hospital patients referred for cognitive screening. Combining latent profile analysis, Gaussian graphical modeling, and exploratory Bayesian DAG analysis, the study produced five principal findings: (a) cognitive and affective domains formed two nearly completely separated network communities; (b) the exploratory directional structures differed between profiles in which cross-domain edges appeared, not in the direction of any given edge; (c) three qualitatively distinct patient subtypes that challenge a simple severity continuum; (d) HD_F1 and LANG as primary cross-domain bridge nodes; and (e) exploratory network comparison indicated differences between Profiles 2 and 3 in global strength and structure, with no edge-level difference surviving correction for multiple comparisons.

### 4.1 Cognitive and affective domains formed two nearly isolated communities

The most striking structural feature of the overall network was the near-complete separation between cognitive and affective nodes. Both community detection algorithms recovered the *a priori* two-community partition perfectly (NMI = 1.000), and the mean absolute cross-domain correlation was only 0.058, compared with 0.495 within the cognitive community and 0.359 within the affective community. Only 13 of the retained edges crossed the domain boundary, with the strongest at just 0.078.

This degree of separation exceeds what has been reported in prior cognitive-affective network studies. Harlev et al. (9) also observed a two-community structure using ACE-R cognitive domains and HADS affective symptoms, but their network retained substantially stronger cross-domain connections, with bridge symptoms shifting from dysphoria in younger adults to anhedonia in older adults. Three features of our study may explain why the separation was stronger.

First, the affective instruments in the present study were clinician-rated scales (HAMA and HAMD-24) rather than self-report measures. When patients complete both cognitive and affective questionnaires themselves, shared response tendencies can inflate cross-domain correlations. By using clinician-rated affective assessment alongside objective cognitive testing, the present design reduced this source of shared variance.

Second, the constructs measured by MoCA domains and HAMA/HAMD-24 factors are more conceptually distant than those captured by self-report batteries. MoCA domains index objective cognitive ability, whereas HAMA/HAMD-24 factors reflect clinician-observed symptom severity. This is a wider gap than between self-reported cognitive complaints and self-reported mood, which tend to share a common subjective appraisal process.

Third, over half of the sample (53.6%) scored below the MoCA cutoff. Patients with substantial cognitive difficulty may not feel anxious or depressed about it, especially when they are unaware of it. This separation was unchanged after adjustment for age and education and in the subgroup aged 60 years or older (Section 3.8).

### 4.2 Directional structures differed between profiles in which edges appeared, not in direction

Whether lower cognitive performance leads to anxiety and depression, or emotional disturbance impairs cognitive performance, is a longstanding question (6). As noted in the Introduction, depression is a risk factor for dementia and is considered modifiable (4, 5), so the direction of the relationship matters for prevention.

The original version of this study interpreted the DAG results as a Simpson’s paradox, on the grounds that the aggregate DAG contained more cognitive-to-affective than affective-to-cognitive edges and the profile-specific DAGs did not. On re-examination, this interpretation was not justified. A Simpson’s paradox concerns a single relationship whose direction differs between the aggregate and its subgroups (29). Our comparison counted different edges across separately estimated graphs, and when the edges are compared one by one, no cross-domain edge was stable in two samples with opposite directions. The samples differed in which cross-domain edges appeared and how many. The edge counts in the original version were also taken from point-estimate graphs; under bootstrap, three of the seven aggregate cross-domain edges and both of the Profile 3 cross-domain edges were unstable.

What remains is more modest. One cross-domain edge, ORI to HD_F5, was directed from cognitive to affective nodes in every sample in which it appeared, before and after adjustment for age and education. LANG to HD_F1 ran in the same direction in the overall sample and in Profile 2, before and after adjustment; after adjustment, an edge between HD_F1 and LANG also appeared in Profile 3 with the opposite majority direction, but its direction certainty of 0.55 means that it is undetermined rather than reversed (Section 3.8). Most of the remaining cross-domain edges had direction certainty close to 0.50 or were present in only one sample. Although most cross-domain edges were compelled within their equivalence classes, identifiability within the class depends on the absence of unmeasured confounders, on linearity, and on faithfulness, and bootstrap stability indicates reproducibility rather than correctness. The aggregate edge set was also sensitive to the algorithm (Section 3.7). The directed structures reported here are therefore exploratory and generate hypotheses for longitudinal studies rather than establishing temporal sequences.

The differences between profiles also have explanations that do not involve different mechanisms. The profiles were formed from the same indicators used in the networks, so within-profile variability was restricted, most strongly for cognitive indicators in Profiles 2 and 3 and for both domains in Profile 3, the only profile with no stable cross-domain edge (Section 4.6). The profiles also differ in size (114, 167, and 267), which affects how many edges are detected. Profile 1, the smallest, had the most cross-domain edges and the lowest direction certainty, consistent with instability at small *n*. The number of stable cross-domain edges did not follow sample size (six, three, and none in Profiles 1, 2, and 3), so sample size alone does not explain the pattern, but it does affect the reliability of each profile-specific graph, and the profile-specific structures were not compared statistically.

McNally et al. (12) combined GGM with Bayesian DAG in bipolar disorder without examining population heterogeneity. Our results suggest that heterogeneity should be examined before an aggregate directional structure is interpreted, because the edges present in the aggregate graph were not all present within its subgroups. They do not show that the direction of the cognitive-affective relationship differs between subgroups.

### 4.3 Three qualitatively distinct subtypes challenge the severity continuum

A common clinical assumption is that cognitive and emotional problems move in tandem: the worse the cognition, the worse the mood. The latent profile analysis challenges that view. Profile 1 patients had very low cognitive performance (mean MoCA = 10.5, all below the cutoff) but only moderate affective symptoms. Profile 2 patients had a mean MoCA of 24.4 and the highest affective burden in the sample (HAMA = 20.7, HAMD-24 = 23.6). Profile 3 had a similar mean MoCA (25.5) and the lowest affective scores. MMSE external validation confirmed that Profiles 2 and 3 did not differ on cognitive performance (*p* = 0.160) despite sharply different emotional profiles. These are qualitatively different clinical presentations, not points along a severity gradient.

Age and education are a partial alternative explanation for this structure. Profile 1 was on average 12 years older than the other profiles and included most of the participants with no formal education, and a multinomial model of age and education classified participants into profiles better than chance. However, the same model did not distinguish Profile 2 from Profile 3, whose difference lies in affective burden with similar cognitive scores. The profiles therefore appear to reflect two separable sources of heterogeneity: an age- and education-related gradient in cognitive performance, which largely defines Profile 1, and a difference in affective burden that is independent of age and education, which separates Profiles 2 and 3. Profile 1 should be interpreted with this overlap in mind, and clinical characterization of this group is needed before it can be treated as a distinct clinical entity.

The exploratory network comparison test was consistent with this: it indicated differences between Profiles 2 and 3 in global strength (*p* = 0.034) and network structure (*p* = 0.014), and the two edges with uncorrected *p* < 0.05 were both within the affective community, although neither survived correction for multiple comparisons. This suggests, without establishing, that the structural contrast between the two profiles lies in how affective symptoms interconnect, consistent with evidence that symptom networks can differ substantially in density across subgroups even when mean levels are similar (8). One finding specific to Profile 3 was that MEM (delayed recall) rose to the second-highest expected influence, a pattern absent in other profiles. In the low affective burden profile, memory concern may prompt clinical help-seeking rather than reflect lower objective performance, consistent with the observation that subjective memory complaints in community-dwelling older adults are more closely linked to anxiety and depression than to objective cognitive performance (30).

### 4.4 Bridge nodes connecting cognition and affect

HD_F1 (anxiety/somatization) and LANG (language) emerged as the two primary bridge nodes linking the cognitive and affective communities, connected by the strongest cross-domain edge in the network (regularized partial correlation = 0.078). This finding contrasts with Zhang et al. (10), who identified sad mood as the key bridge between depressive symptoms and cognitive performance using the self-report PHQ-9 and MoCA-5min. The difference likely reflects the measurement source. Self-report instruments capture subjective emotional experience, making mood-related items natural bridge candidates. Clinician-rated instruments such as the HAMD-24 instead emphasize observable signs, which may explain why the somatic manifestation of anxiety rather than subjective sadness surfaced as the affective-side bridge in the present study. This interpretation aligns with evidence that clinician-rated and self-report depression networks produce significantly different community structures and edge configurations (31). The identity of the bridge nodes was less robust than the community structure. After adjustment for age and education, HD_F1 and LANG remained among the top three bridge nodes but HD_F4 rose to the highest bridge expected influence and separated from the main affective community, and in the subgroup aged 60 years or older the bridge ordering was too unstable to interpret (Section 3.8). The bridge findings should therefore be read as candidates for replication rather than established results.

The bridge node findings and the DAG findings address different questions. Bridge expected influence indexes the structural position of nodes that carry cross-domain associations in the undirected network, regardless of direction. The DAGs describe which cross-domain edges appeared in each sample and how certain their direction was. The two analyses agree on one point: the edge between LANG and HD_F1, which connected the two primary bridge nodes, also ran from cognitive to affective nodes in the overall sample and in Profile 2, before and after adjustment, although its direction certainty in the overall sample was modest (0.58 unadjusted, 0.65 adjusted) and, after adjustment, an edge between the two nodes appeared in Profile 3 with undetermined direction (Section 3.8). Whether HD_F1 or LANG is a useful target for intervention cannot be decided from these data, because the direction of most cross-domain edges was undetermined and the design is cross-sectional (Section 4.6).

### 4.5 Clinical implications

The findings have three implications for cognitive screening clinics, all of which concern assessment rather than the order of treatment.

First, the near-complete separation between the cognitive and affective communities (NMI = 1.000, mean cross-domain |*r*| = 0.058) argues against the assumption that improving one domain will automatically benefit the other. A patient whose MoCA score improves after cognitive training cannot be assumed to feel less anxious, and a patient whose depression remits cannot be assumed to think more clearly. Each domain requires its own assessment, its own monitoring, and its own outcome benchmarks.

Second, the three profiles describe different clinical presentations that a total score would not distinguish. Profile 1 patients were older (mean age 68 years) and had markedly lower cognitive performance with moderate affective symptoms, so affective symptoms should be assessed in this group and not assumed to be absent because cognition is the presenting problem. Profile 2 patients had the highest affective burden in the sample with cognitive scores similar to Profile 3, so affective burden cannot be inferred from cognitive performance and requires its own assessment. Profile 3 patients had mild symptoms on both dimensions, and MEM was unusually prominent in their network, which suggests that memory concern may be a common reason for referral in this group; psychoeducation on the distinction between age-related memory changes and impairment that warrants further evaluation, with periodic follow-up, may be more appropriate than immediate intervention, because overmedicalization of memory worry in cognitively intact adults carries its own risks (30). Whether any profile benefits from treating one domain before the other cannot be determined from these data; the directional structures were exploratory, most cross-domain edges had undetermined direction, and a treatment sequence would need longitudinal or interventional studies.

Third, HD_F1 (anxiety/somatization) and LANG (language) were the nodes through which the two communities were connected. They mark where cross-domain associations were concentrated in this sample and are candidates for closer assessment and for future intervention studies. They are not confirmed mechanisms. The edge between them ran from LANG to HD_F1 in the overall sample and in Profile 2, before and after adjustment for age and education, with modest direction certainty in the overall sample (0.58 unadjusted, 0.65 adjusted); after adjustment, an edge between the two nodes also appeared in Profile 3, with undetermined direction (Section 3.8). The data therefore do not show that affective disturbance impairs language, and no direction of effect should be assumed in practice.

The results also carry a methodological point. Directional structures estimated in a mixed clinical population were not all reproduced within its subgroups, so studies of the cognitive-affective relationship in such populations should examine subgroup heterogeneity before interpreting aggregate directional findings.

### 4.6 Limitations

Several limitations should be considered. First, the cross-sectional design means that all DAG-derived directions are exploratory hypotheses, not established temporal sequences. Longitudinal replication with repeated assessments is needed to test whether the two edges that ran from cognitive to affective nodes where their direction was determined, ORI to HD_F5 and LANG to HD_F1, hold over time. Second, data were drawn from the assessment service of a single general hospital, which receives referrals mainly from the sleep medicine center, neurology, and geriatrics, limiting generalizability to other clinical settings and populations. In addition, the sample spanned the full adult age range (18–89 years); the findings therefore characterize a cognitive screening population rather than any specific age stratum and should not be extrapolated to older adults, or to any other age group, without replication in age-defined samples. The adjustment for age and education (Section 3.8) was a linear residualization on two measured covariates; it does not rule out nonlinear effects of these variables or confounding by unmeasured variables.

Third, the assessment system did not record reasons for referral, psychiatric or neurological diagnoses, medical comorbidities, sleep disorders, or current medications, and these could not be retrieved from clinical records. This limits interpretation in several ways. Part of the sample was referred from a sleep medicine center, so sleep disorders are likely to be common; they can affect both cognitive and affective scores and may account for part of the association between them. Medication status is a particular concern. The mean HAMA and HAMD-24 scores were at levels at which anxiolytic or antidepressant treatment is commonly indicated, and higher still in Profile 2, so a substantial part of the sample was probably being treated at the time of assessment. Affective scores measured under treatment are lower than they would otherwise be, which affects the estimated profile boundaries and could place treated patients in the low affective burden profile; benzodiazepines, sedating antidepressants, and drugs with anticholinergic effects can reduce performance on attention, memory, and other MoCA domains, which could create or strengthen cross-domain associations; and if treatment status differed between profiles, some profile differences in cross-domain edges could reflect treatment rather than the cognitive-affective relationship. None of this could be examined. Profile 1, with a mean MoCA of 10.5, is likely to include patients with dementia, post-stroke cognitive impairment, or other neurological conditions that we could not identify, so this profile should not be read as a single clinical entity. The associations we report may therefore partly reflect unmeasured clinical factors, and the profiles and cross-domain structures need to be examined in samples with full clinical characterization. In prospective studies, medication status at the time of assessment should be recorded as a core variable and verified against clinical records, so that the cognitive-affective network can be estimated separately in treated and untreated patients.

Fourth, the bridge node findings are the least stable results in this study. Profile-specific bridge expected influence could not be computed because the within-profile networks were too sparse; the 14-node and 15-node sensitivity models met the same limitation; floor effects in Profile 1 and ceiling effects in several MoCA domains (NAM 78.6%, ORI 64.8%, LANG 61.9%, ABS 59.5%) may have reduced cross-domain edge strength; and although the CS-coefficient for bridge expected influence in the primary model (0.361) exceeded the acceptable threshold, the bridge ordering changed after adjustment for age and education and was uninterpretable in the older subgroup (CS-coefficient 0.051). Bridge node findings should therefore be regarded as preliminary and sensitive to sample composition.

Fifth, the DAG results are exploratory. Gaussian BIC is score-equivalent, so the direction of an edge is identifiable only through v-structures within the Markov equivalence class and depends on the assumptions of no unmeasured confounding, linearity, and faithfulness. Bootstrap stability indicates reproducibility rather than correctness, and direction certainty was close to 0.50 for most cross-domain edges. The point-estimate graphs depended on the search algorithm, mainly in edges with low bootstrap inclusion or direction certainty close to 0.50 (Section 3.7), and the number of cross-domain edges was small (seven, six, three, and two), so counts and ratios of edges were not tested statistically and are no longer interpreted. Profile-specific DAGs are further affected by restricted within-profile variability and by sample size. Directed edges should be read as hypotheses. Sixth, the LPA used the most parsimonious covariance specification (equal variances, zero covariances), and the MoCA education correction followed only the standard procedure of adding one point for 12 years of education or less. An alternative specification with equal within-profile covariances fitted slightly better by BIC and reassigned 30.3% of participants, mostly between Profiles 2 and 3, so the boundary between these two profiles is partly dependent on the covariance specification. In addition, ten of the sixteen indicators take fewer than ten distinct values, which departs from the continuous measurement level assumed by latent profile analysis and limited the range of specifications that could be estimated. Cognitive status was characterized by the MoCA alone. Clinical history, functional assessment, and a clinical diagnostic evaluation were not available, so no participant was diagnosed with mild cognitive impairment, and scores below the cutoff should be read as low performance on a screening instrument rather than as a diagnosis. MMSE scores were available for 69.7% of participants; their availability was unrelated to cognitive performance and profile membership, but participants with MMSE data had slightly higher affective scores, so the external validation should be read with this in mind.

Seventh, the profiles were derived from the same 16 indicators that were then used to estimate the profile-specific networks. This has two consequences. Profile membership was treated as known although it is estimated; average posterior probabilities were high (0.965, 0.929, and 0.958), but the profile-specific networks do not carry forward the uncertainty of assignment, which is largest at the boundary between Profiles 2 and 3. More importantly, latent profile analysis separates participants on the means of the indicators, so within-profile variability is restricted, partial correlations are attenuated, and edges are lost. The within-profile standard deviations of the cognitive indicators in Profiles 2 and 3 were about 60% of the full-sample values, and those of the affective indicators in Profile 3 about 66%; Profile 1 retained most of the cognitive variability but its cognitive scores were concentrated near the floor. The sparse within-profile networks, the expected influence of zero for five of the seven cognitive nodes in Profile 1, and the absence of any stable cross-domain edge in Profile 3, the only profile in which both domains were restricted, are all consistent with these patterns. Differences between within-profile and overall networks should therefore not be interpreted as evidence that the structure of the cognitive-affective relationship differs between profiles, and the profile-specific directed structures are presented as exploratory for this reason. The network comparison test is subject to the same caution: the comparisons involving Profile 1 were underpowered, the global tests were not corrected for multiple comparisons, and none of the 120 edge-level differences between Profiles 2 and 3 survived correction, so these results are also exploratory.

## 5 Conclusion

In 548 Chinese general hospital patients referred for cognitive screening, MoCA cognitive domains and clinician-rated HAMA and HAMD-24 affective factors formed two nearly completely separated network communities (NMI = 1.000), connected mainly through LANG and HD_F1. Latent profile analysis identified three distinct profiles that did not align with a simple severity continuum. Exploratory directed acyclic graph analysis found few stable cross-domain edges, most with undetermined direction; the two edges with the highest direction certainty ran from cognitive to affective nodes where their direction was determined, and the profiles differed in which edges appeared rather than in their direction. These findings support stratified assessment in cognitive screening settings and indicate that directional structures estimated in a heterogeneous population should be examined within its subgroups before they are interpreted. They do not establish a causal direction or a treatment sequence.

## Data Availability

The raw data supporting the conclusions of this article will be made available by the authors, without undue reservation.
