# Supplementary material for: Cognitive and affective networks in patients referred for cognitive screening: latent profiles, bridge nodes, and exploratory directional structures
Source: Front Psychiatry. 2026 Oct 8;17:1887588. doi: 10.3389/fpsyt.2026.1887588 (PMC13687087; doi:10.3389/fpsyt.2026.1887588)
Supplement: Supplementary file 1 [file SupplementaryFile1.docx]

**Supplementary Table S1.** Fit indices, profile sizes, and classification quality for latent profile solutions with one to six profiles.

| **k** | **Log-likelihood** | **Par.** | **AIC** | **BIC** | **SABIC** | **Entropy** | **BLRT p** | **Profile sizes, n** | **Average posterior probability** |
| --- | --- | --- | --- | --- | --- | --- | --- | --- | --- |
| 1 | −16205.06 | 32 | 32474.12 | 32611.92 | 32510.34 | 1.000 |  | 548 | 1.000 |
| 2 | −15641.42 | 49 | 31380.83 | 31591.84 | 31436.29 | 0.845 | 0.0099 | 208 / 340 | 0.934 / 0.966 |
| 3 | −14887.19 | 66 | 29906.38 | 30190.60 | 29981.08 | 0.891 | 0.0099 | 114 / 167 / 267 | 0.965 / 0.929 / 0.958 |
| 4 | −14699.58 | 83 | 29565.16 | 29922.58 | 29659.10 | 0.915 | 0.0099 | 94 / 194 / 238 / 22 | 0.977 / 0.941 / 0.953 / 0.980 |
| 5 | −14389.31 | 100 | 28978.61 | 29409.24 | 29091.80 | 0.919 | 0.0099 | 43 / 168 / 205 / 107 / 25 | 0.978 / 0.935 / 0.950 / 0.945 / 0.951 |
| 6 | −14242.27 | 117 | 28718.54 | 29222.37 | 28850.96 | 0.928 | 0.0099 | 38 / 168 / 32 / 202 / 87 / 21 | 0.969 / 0.943 / 0.992 / 0.953 / 0.927 / 0.934 |

Note. All models were estimated with tidyLPA (mclust EEI parameterization: equal variances across profiles, covariances fixed to zero) on the 16 indicators on their original scales, n = 548. Par. = number of free parameters. SABIC = sample-size adjusted BIC. BLRT = bootstrap likelihood ratio test comparing k with k − 1 profiles, 100 bootstrap replications; p = 0.0099 is the smallest value obtainable with 100 replications, and the test was significant at every step. Average posterior probability = mean of the posterior probability of the assigned profile among participants assigned to that profile. Profiles are listed in the order returned by the software. Decreases in BIC between successive solutions: 1020.08, 1401.24, 268.02, 513.34, and 186.87. From four profiles onward, the smallest profile contained fewer than 5% of the sample (22, 25, and 21 participants). The three-profile solution was retained (see Section 3.2).

**Supplementary Table S2.** Comparison of covariance specifications for the three-profile solution, and cross-classification of participants under the retained and the alternative specification.

**A. Fit of the three-profile model under four covariance specifications**

| **Specification (mclust)** | **Par.** | **Log-likelihood** | **AIC** | **BIC** | **CAIC** | **AWE** | **Entropy** | **Average posterior probability, min / max** | **Smallest / largest profile, %** |
| --- | --- | --- | --- | --- | --- | --- | --- | --- | --- |
| Model 1: equal variances, zero covariances (EEI); retained | 66 | −14887.19 | 29906.38 | 30190.60 | 30256.60 | 30803.03 | 0.891 | 0.933 / 0.972 | 20.8 / 48.7 |
| Model 2: free variances, zero covariances (VVI) |  | not estimable |  |  |  |  |  |  |  |
| Model 3: equal variances, equal covariances (EEE) | 306 | −14079.29 | 28770.57 | 30088.29 | 30394.29 | 32934.29 | 0.861 | 0.934 / 0.967 | 28.3 / 40.9 |
| Model 6: free variances, free covariances (VVV) |  | not estimable |  |  |  |  |  |  |  |

**B. Cross-classification of participants: Model 1 profiles (rows) by Model 3 profiles (columns)**

| **Model 1 profile** | **Model 3 profile 1** | **Model 3 profile 2** | **Model 3 profile 3** | **Total** | **Agreement with Model 3, %** |
| --- | --- | --- | --- | --- | --- |
| Profile 1 (low cognitive performance) | 101 | 3 | 10 | 114 | 88.6 |
| Profile 2 (high affective burden) | 20 | 107 | 40 | 167 | 64.1 |
| Profile 3 (low affective burden) | 34 | 59 | 174 | 267 | 65.2 |
| Total | 155 | 169 | 224 | 548 | 69.7 |

Note. All models were fitted with tidyLPA to the 16 indicators on their original scales, n = 548, three profiles. Par. = number of free parameters. Models 2 and 6 could not be estimated by mclust with 16 indicators and three profiles. Model 3 had a lower BIC than Model 1 (difference 102.3) but required 306 parameters, had a higher CAIC and AWE, lower entropy, and a different profile structure: 30.3% of participants were assigned to a different profile, mostly across the boundary between Profiles 2 and 3. Model 3 profiles are numbered in the order returned by the software.

**Supplementary Table S3.** Cross-domain directed edges in the directed acyclic graphs estimated on the unadjusted data, for the overall sample and each profile.

| **Sample** | **Edge** | **Direction** | **Inclusion** | **Direction certainty** | **In point estimate** | **Compelled in CPDAG** |
| --- | --- | --- | --- | --- | --- | --- |
| Overall sample (n = 548) | ORI → HD_F5 | Cognitive → Affective | 0.998 | 0.80 | Yes | Yes |
|  | LANG → HD_F1 | Cognitive → Affective | 0.687 | 0.58 | No | n.a. |
|  | VE → HD_F4 | Cognitive → Affective | 0.683 | 0.58 | Reversed | Yes |
|  | ORI → HD_F1 | Cognitive → Affective | 0.664 | 0.52 | Yes | Yes |
|  | ORI → HD_F6 | Cognitive → Affective | 0.584 | 0.71 | Yes | No |
|  | HD_F1 → VE | Affective → Cognitive | 0.478 | 0.82 | Yes | Yes |
|  | HA_P → NAM | Affective → Cognitive | 0.471 | 0.61 | Reversed | Yes |
|  | HA_S → ORI | Affective → Cognitive | 0.468 | 0.75 | Reversed | Yes |
| Profile 1 (n = 114) | NAM → HA_P | Cognitive → Affective | 0.840 | 0.56 | Reversed | Yes |
|  | ORI → HD_F5 | Cognitive → Affective | 0.830 | 0.64 | Yes | Yes |
|  | ORI → HD_F6 | Cognitive → Affective | 0.577 | 0.61 | Yes | Yes |
|  | HD_F1 → ORI | Affective → Cognitive | 0.538 | 0.51 | No | n.a. |
|  | ATT → HD_F5 | Cognitive → Affective | 0.528 | 0.58 | No | n.a. |
|  | VE → HA_P | Cognitive → Affective | 0.500 | 0.63 | Yes | Yes |
|  | ABS → HD_F5 | Cognitive → Affective | 0.456 | 0.54 | Reversed | Yes |
|  | HA_S → ORI | Affective → Cognitive | 0.455 | 0.79 | Yes | Yes |
| Profile 2 (n = 167) | LANG → HD_F1 | Cognitive → Affective | 0.689 | 0.77 | Yes | Yes |
|  | ATT → HD_F5 | Cognitive → Affective | 0.597 | 0.52 | Reversed | Yes |
|  | HD_F5 → NAM | Affective → Cognitive | 0.569 | 0.59 | Yes | Yes |
| Profile 3 (n = 267) | HD_F4 → ATT | Affective → Cognitive | 0.440 | 0.80 | Yes | Yes |
|  | HD_F1 → VE | Affective → Cognitive | 0.402 | 0.62 | Yes | Yes |

Note. DAGs were learned with hill-climbing (BIC score) on the 16 indicators and 1,000 nonparametric bootstrap replications; the same procedure was applied to the overall sample and to each profile separately. The table lists every cross-domain edge (linking a cognitive and an affective node) that appeared in the point-estimate DAG or reached bootstrap inclusion of 0.50. Inclusion is the proportion of bootstrap networks containing the edge in either direction. Edges are listed in the majority bootstrap direction, and direction certainty is the proportion of those networks in which the edge ran in the direction shown; values near 0.50 indicate that the direction was undetermined. In point estimate: whether the edge appears in the single DAG learned from the full data in the direction shown (Yes), appears with the opposite direction (Reversed), or does not appear (No). Compelled in CPDAG: whether the arc in the point-estimate DAG was compelled in the completed partially directed acyclic graph (its direction fixed by v-structures) or reversible within the equivalence class; n.a. = not in the point-estimate DAG. Cross-domain arcs in the point-estimate DAGs: overall, 7 arcs (ORI → HD_F6, ORI → HD_F5, ORI → HD_F1, ORI → HA_S, NAM → HA_P, HD_F1 → VE, HD_F4 → VE), of which 6 were compelled; Profile 1, 6 arcs (ORI → HD_F5, ORI → HD_F6, VE → HA_P, HA_P → NAM, HD_F5 → ABS, HA_S → ORI), all compelled; Profile 2, 3 arcs (LANG → HD_F1, HD_F5 → ATT, HD_F5 → NAM), all compelled; Profile 3, 2 arcs (HD_F1 → VE, HD_F4 → ATT), both compelled. Results after adjustment for age and education are given in Supplementary Table S5.

**Supplementary Table S4.** Expected influence (EI) and bridge expected influence (bEI) in the main network and after adjustment for age and education.

| **Node** | **Community** | **EI, main** | **EI, adjusted** | **bEI, main** | **bEI, adjusted** |
| --- | --- | --- | --- | --- | --- |
| HA_P | Affective | 1.127 (1) | 1.063 (2) | −0.029 | −0.087 |
| HD_F3 | Affective | 1.036 (2) | 1.117 (1) | −0.034 | 0.000 |
| ATT | Cognitive | 1.006 (3) | 0.936 (4) | 0.000 | −0.027 |
| HD_F1 | Affective | 0.975 (4) | 0.912 (5) | +0.109 (1) | +0.101 (3) |
| LANG | Cognitive | 0.962 (5) | 1.004 (3) | +0.086 (2) | +0.105 (2) |
| VE | Cognitive | 0.852 | 0.711 | −0.064 | −0.097 |
| ABS | Cognitive | 0.794 | 0.736 | 0.000 | +0.014 |
| HA_S | Affective | 0.792 | 0.812 | −0.008 | +0.030 |
| MEM | Cognitive | 0.754 | 0.453 | 0.000 | +0.007 |
| HD_F7 | Affective | 0.751 | 0.728 | −0.037 | −0.091 |
| HD_F5 | Affective | 0.654 | 0.608 | −0.051 | −0.093 |
| ORI | Cognitive | 0.647 | 0.776 | +0.001 | +0.084 |
| NAM | Cognitive | 0.645 | 0.667 | −0.037 | +0.033 |
| HD_F4 | Affective | 0.551 | 0.800 | +0.026 | +0.159 (1) |
| HD_F6 | Affective | 0.406 | 0.420 | +0.010 | +0.076 |
| HD_F2 | Affective | 0.245 | 0.355 | 0.000 | +0.024 |
| CS-coefficient |  | 0.750 | 0.750 | 0.361 | 0.284 |
| Cross-domain edges |  | 13 | 19 |  |  |
| Strongest cross-domain edge |  | LANG–HD_F1 (0.078) | LANG–HD_F1 (0.080) |  |  |
| Community detection NMI (walktrap / spinglass) |  | 1.000 / 1.000 | 0.821 / 0.821 |  |  |

Note. Main network: n = 548, Spearman correlations, EBICglasso with γ = 0.5. Adjusted network: n = 543 (five participants with missing education excluded); each of the 16 indicators was standardized and regressed on age and education (six ordinal levels), and the residuals were used to re-estimate the network with the same procedure. Ranks in parentheses are given for the five nodes with the highest EI in the main network and for the three nodes with the highest positive bEI. CS-coefficient = correlation stability coefficient from a case-dropping bootstrap (1000 replications). NMI = normalized mutual information between the data-driven community partition and the a priori two-community partition; in the adjusted network both algorithms kept all cognitive nodes in one community and all affective nodes apart from the cognitive nodes, but placed HD_F2 (weight) and HD_F4 (diurnal variation) in a separate small community within the affective side. The adjusted model selected a lower EBIC penalty than the main model, so its weaker edges should be interpreted with caution.

**Supplementary Table S5.** Cross-domain directed edges in the directed acyclic graphs estimated on age- and education-adjusted data, for the overall sample and each profile.

| **Sample** | **Edge** | **Direction** | **Inclusion** | **Direction certainty** | **In point estimate** |
| --- | --- | --- | --- | --- | --- |
| Overall sample (n = 543) | ORI → HD_F5 | Cognitive → Affective | 0.996 | 0.81 | Yes |
|  | VE → HD_F4 | Cognitive → Affective | 0.824 | 0.79 | Yes |
|  | LANG → HD_F1 | Cognitive → Affective | 0.766 | 0.65 | Yes |
|  | ORI → HD_F6 | Cognitive → Affective | 0.644 | 0.81 | Reversed |
|  | ORI → HD_F1 | Cognitive → Affective | 0.602 | 0.54 | Yes |
|  | HA_S → ORI | Affective → Cognitive | 0.510 | 0.68 | No |
| Profile 1 (n = 113) | ORI → HD_F5 | Cognitive → Affective | 0.886 | 0.65 | Yes |
|  | HA_P → NAM | Affective → Cognitive | 0.714 | 0.61 | Yes |
|  | ORI → HD_F6 | Cognitive → Affective | 0.616 | 0.67 | Yes |
|  | VE → HA_P | Cognitive → Affective | 0.598 | 0.63 | Yes |
|  | HD_F5 → VE | Affective → Cognitive | 0.544 | 0.52 | No |
|  | ABS → HD_F3 | Cognitive → Affective | 0.514 | 0.53 | No |
|  | ABS → HD_F5 | Cognitive → Affective | 0.504 | 0.54 | Yes |
|  | ATT → HD_F7 | Cognitive → Affective | 0.502 | 0.51 | No |
|  | HD_F1 → ORI | Affective → Cognitive | 0.500 | 0.52 | Yes |
| Profile 2 (n = 166) | ABS → HA_P | Cognitive → Affective | 0.686 | 0.89 | Yes |
|  | LANG → HD_F1 | Cognitive → Affective | 0.556 | 0.70 | Yes |
|  | ABS → HD_F4 | Cognitive → Affective | 0.508 | 0.51 | No |
| Profile 3 (n = 264) | HD_F4 → VE | Affective → Cognitive | 0.682 | 0.62 | Yes |
|  | HD_F1 → LANG | Affective → Cognitive | 0.540 | 0.55 | Yes |

Note. Each of the 16 indicators was standardized and regressed on age and education (six ordinal levels); DAGs were learned on the residuals with hill-climbing (BIC score) and 500 nonparametric bootstrap replications, using the same procedure as the main analysis. Five participants with missing education were excluded. Edges are listed when bootstrap inclusion (the proportion of bootstrap networks containing the edge in either direction) was at least 0.50, in the majority direction; direction certainty is the proportion of those networks in which the edge ran in the direction shown. In point estimate: whether the edge appears in the single DAG learned from the full adjusted data (Yes), appears with the opposite direction (Reversed), or does not appear (No). Cross-domain arcs in the point-estimate DAGs: overall, 9 arcs (ORI → HD_F5, HD_F1 → VE, NAM → HA_P, VE → HD_F4, LANG → HD_F1, HD_F6 → ORI, ABS → HA_S, ORI → HD_F1, ATT → HA_P), of which 8 were compelled; Profile 1, 8 arcs (ABS → HD_F5, ORI → HD_F6, VE → HA_P, ORI → HD_F5, HD_F1 → ORI, HA_P → NAM, MEM → HD_F6, ATT → HD_F3), 7 compelled; Profile 2, 2 arcs (LANG → HD_F1, ABS → HA_P), both compelled; Profile 3, 3 arcs (HD_F1 → LANG, HD_F4 → VE, ABS → HA_S), all compelled. Edges with direction certainty near 0.50 should be regarded as undetermined in direction.

**Supplementary Table S6.** Expected influence (EI) and bridge expected influence (bEI) in the full sample and in the subgroup aged 60 years or older.

| **Node** | **Community** | **EI, full sample** | **EI, ≥60 years** | **bEI, full sample** | **bEI, ≥60 years** |
| --- | --- | --- | --- | --- | --- |
| HA_P | Affective | 1.127 (1) | 0.945 (2) | −0.029 | −0.063 (2) |
| HD_F3 | Affective | 1.036 (2) | 0.987 (1) | −0.034 | 0.000 |
| ATT | Cognitive | 1.006 (3) | 0.931 (3) | 0.000 | −0.030 |
| HD_F1 | Affective | 0.975 (4) | 0.827 (5) | +0.109 (1) | +0.076 (1) |
| LANG | Cognitive | 0.962 (5) | 0.917 (4) | +0.086 (2) | +0.026 (7) |
| VE | Cognitive | 0.852 | 0.726 | −0.064 (3) | −0.052 (4) |
| ABS | Cognitive | 0.794 | 0.665 | 0.000 | −0.011 |
| HA_S | Affective | 0.792 | 0.652 | −0.008 | 0.000 |
| MEM | Cognitive | 0.754 | 0.616 | 0.000 | 0.000 |
| HD_F7 | Affective | 0.751 | 0.712 | −0.037 | 0.000 |
| HD_F5 | Affective | 0.654 | 0.504 | −0.051 | −0.062 (3) |
| ORI | Cognitive | 0.647 | 0.734 | +0.001 | +0.033 (5) |
| NAM | Cognitive | 0.645 | 0.542 | −0.037 | −0.015 |
| HD_F4 | Affective | 0.551 | 0.308 | +0.026 | 0.000 |
| HD_F6 | Affective | 0.406 | 0.333 | +0.010 | 0.000 |
| HD_F2 | Affective | 0.245 | 0.021 | 0.000 | 0.000 |
| CS-coefficient |  | 0.750 | 0.593 | 0.361 | 0.051 |
| Cross-domain edges |  | 13 | 8 |  |  |
| Strongest cross-domain edge |  | LANG–HD_F1 (0.078) | ORI–HD_F1 (0.050) |  |  |
| Community detection NMI (walktrap / spinglass) |  | 1.000 / 1.000 | 1.000 / 0.875 |  |  |

Note. Subgroup network: n = 275, estimated with the same procedure as the main network (Spearman correlations, EBICglasso with γ = 0.5). Ranks in parentheses are given for the five nodes with the highest EI in the full sample and for the nodes ranked in the top five on bEI in either sample; LANG, second on bEI in the full sample, ranked seventh in the subgroup. CS-coefficient = correlation stability coefficient from a case-dropping bootstrap (1000 replications). The CS-coefficient for bEI in the subgroup (0.051) is below the 0.25 threshold, so the bEI ordering in the subgroup should not be interpreted. NMI = normalized mutual information between the data-driven community partition and the a priori two-community partition. Cross-domain edges in the subgroup network (regularized partial correlations): ORI–HD_F1 0.050; VE–HA_P −0.037; ATT–HD_F5 −0.030; LANG–HD_F1 0.026; ORI–HD_F5 −0.017; NAM–HA_P −0.015; VE–HD_F5 −0.015; ABS–HA_P −0.011.

**Supplementary Table S7.** Comparison of participants with and without MMSE data.

| **Variable** | **With MMSE (n = 382)** | **Without MMSE (n = 166)** | **p** |
| --- | --- | --- | --- |
| Age, years, mean (SD) | 58.41 (14.21) | 57.81 (13.30) | 0.635 |
| Education level (0 to 5), mean (SD) | 2.23 (1.20) | 2.25 (1.09) | 0.799 |
| Sex, male / female, n | 165 / 217 | 74 / 92 | 0.836 |
| MoCA total (education corrected), mean (SD) | 22.86 (6.99) | 22.86 (7.06) | 0.996 |
| HAMA total, mean (SD) | 14.75 (7.92) | 13.19 (6.92) | 0.021 |
| HAMD-24 total, mean (SD) | 16.27 (7.75) | 14.54 (8.07) | 0.021 |
| Profile 1 / 2 / 3, n | 81 / 123 / 178 | 33 / 44 / 89 | 0.287 |

Note. Continuous variables were compared with Welch t tests and categorical variables with chi-square tests. Education level: 0 = illiterate, 1 = primary school, 2 = junior middle school, 3 = senior middle school, 4 = college or university, 5 = graduate; five participants with missing education are excluded from that row. MMSE availability depended on which assessment combination the treating physician ordered (see Section 2.2).
